# Transcriptional Infidelity Promotes Heritable Phenotypic Change in a Bistable Gene Network

**DOI:** 10.1371/journal.pbio.1000044

**Published:** 2009-02-24

**Authors:** Alasdair J. E Gordon, Jennifer A Halliday, Matthew D Blankschien, Philip A Burns, Fumio Yatagai, Christophe Herman

**Affiliations:** 1 Department of Molecular and Human Genetics, Baylor College of Medicine, Houston, Texas, United States of America; 2 Pathology and Tumour Biology, Leeds Institute for Molecular Medicine, St James's University Hospital, Leeds, United Kingdom; 3 Molecular Imaging Program, RIKEN Institute, Wako-shi, Saitama, Japan; 4 Department of Molecular Virology and Microbiology, Baylor College of Medicine, Houston, Texas, United States of America; Johns Hopkins University, United States of America

## Abstract

Bistable epigenetic switches are fundamental for cell fate determination in unicellular and multicellular organisms. Regulatory proteins associated with bistable switches are often present in low numbers and subject to molecular noise. It is becoming clear that noise in gene expression can influence cell fate. Although the origins and consequences of noise have been studied, the stochastic and transient nature of RNA errors during transcription has not been considered in the origin or modeling of noise nor has the capacity for such transient errors in information transfer to generate heritable phenotypic change been discussed. We used a classic bistable memory module to monitor and capture transient RNA errors: the *lac* operon of Escherichia coli comprises an autocatalytic positive feedback loop producing a heritable all-or-none epigenetic switch that is sensitive to molecular noise. Using single-cell analysis, we show that the frequency of epigenetic switching from one expression state to the other is increased when the fidelity of RNA transcription is decreased due to error-prone RNA polymerases or to the absence of auxiliary RNA fidelity factors GreA and GreB (functional analogues of eukaryotic TFIIS*)*. Therefore, transcription infidelity contributes to molecular noise and can effect heritable phenotypic change in genetically identical cells in the same environment. Whereas DNA errors allow genetic space to be explored, RNA errors may allow epigenetic or expression space to be sampled. Thus, RNA infidelity should also be considered in the heritable origin of altered or aberrant cell behaviour.

## Author Summary

Errors in information transfer from DNA to RNA to protein are inevitable and ubiquitous. When errors that occur in DNA are not repaired and become fixed as permanent mutations, they can have heritable phenotypic consequences for cells. In contrast, errors that occur during RNA transcription are considered transient, because the life span of mRNAs and their encoded proteins is thought to be too short to have heritable consequences. Here, we show that transient errors that arise during transcription can cause a heritable phenotypic change within a population of genetically identical Escherichia coli cells growing in the same environment. We used single-cell observation of the bistable *lac* operon (which allows a cell to have two alternate states) in a stable ON or OFF state. We show that the epigenetic-switch frequency from the OFF to ON state is increased when the fidelity of RNA transcription is altered: bacterial strains that contain error-prone RNA polymerases, “RNA mutators,” and strains deficient in auxiliary RNA fidelity factors exhibit an increased epigenetic-switch frequency compared with wild-type strains. Therefore, like DNA mutation, transient stochastic events can also have long-lived heritable consequences for the cell.

## Abstract 3

RNA errors arise frequently during transcription, but with what consequence? New evidence shows that transient RNA errors can cause heritable phenotypic changes when they activate an autocatalytic positive-feedback loop in gene regulation.

## Introduction

Altered proteins can result from errors incurred at any step during information transfer from DNA to protein. Errors in DNA, RNA, and protein synthesis occur at rates of, very roughly, 10^−9^, 10^−5^, and 10^−4^ errors per residue, respectively [1]. Although rare, errors in DNA synthesis can be fixed as permanent errors—mutations—which can generate heritable change in cellular phenotype. Transcription and translation errors occur more frequently, but are considered transient and their effects fleeting, since the altered molecules are present for a limited time. It has been shown that transcription over a damaged DNA template can generate altered proteins in nondividing DNA repair–deficient cells [2], and it has been suggested that transient errors can produce transient mutators, thereby generating phenotypic change by introducing mutations [3,4]. However, the capacity for transient errors to generate heritable epigenetic phenotypic change has not been considered.

The stochastic nature of gene expression results in random fluctuations in protein numbers per cell [5,6]. Theoretical and experimental studies have culminated in stochastic chemical kinetic models that describe the statistics of molecular noise [7–9]. Many aspects of gene expression have been considered, including rates of transcription and translation and rates of destruction of the corresponding mRNA and protein products. These models address protein quantity; the quality of the protein produced is not considered with transcription and translation deemed error-free processes. However, due to RNA transcription errors, approximately 1% of all mRNAs encoding polypeptides of 300 amino acids will encode erroneous messages [3]. It has been shown in bacteria, yeast, and mammalian cells that gene expression, and the accompanying noise, occurs in stochastic bursts dominated by the production of mRNAs [10–12]. Since one mRNA is translated many times, RNA errors become amplified, challenging the cell with erroneous proteins that may exhibit partial function, loss-of-function, gain-of-function, or dominant-negative properties. Therefore, any cell at any time may be transiently impaired for a function encoded in a rarely made transcript [3].

As first suggested by Delbrück [13], epigenetic differences can be understood in terms of multistability: a given cell can persist in one of many stable steady states, which differ from each other by the genes that are ON and those that are OFF. This multistable nature of biological switches is fundamental for the determination of cell fate in unicellular and multicellular organisms [14–21]. Bistability can arise in gene networks that contain a positive-feedback loop [15]. Such gene networks are often regulated by transcription factors that are present in low abundance and therefore subject to noise [22–26]. The *lac* operon, a set of coordinately expressed genes under the negative control of the *lac* repressor, is a classic bistable gene network with stable ON and OFF states [14,27]. We determined the contribution of RNA errors to molecular noise using a biologically relevant context to monitor noise, namely, heritable stochastic switching in the bistable *lac* gene network.

## Results and Discussion

### Bistability, Hysteresis, and Maintenance in the *lac* System

**Figure 1:**
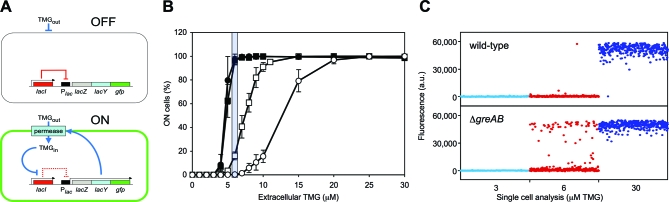
Stochastic Switching in the *lac* Bistable Gene Network (A) Under maintenance conditions, the *lac* operon is OFF when the *lac* repressor is bound to the *lac* operator (indicated by the solid red line) and the inducer TMG remains extracellular; stochastic events that lead to a transient derepression of the *lac* operon will result in a burst of *lac* operon functions and the appearance of permease will initiate an autocatalytic positive-feedback response (indicated by solid blue lines), which will heritably maintain the ON state (TMG induces an allosteric transition in *lac* repressor, indicated by the dashed red line, so that it no longer binds to the *lac* operator), and the cell will exhibit green fluorescence. (B) Cells that were originally ON (filled symbols) or OFF (open symbols) were sub-cultured and grown in media containing various concentrations of TMG. Circles denote the wild-type strain; squares denote the Δ*greA* Δ*greB* strain. The shaded region shows the maintenance concentration of TMG. Each value is the average ± SD from four to six independent cultures. (C) Uninduced (OFF) wild-type (top panel) and Δ*greA* Δ*greB* cells (bottom panel) were diluted and grown in media containing 3 μM (light blue), 6 μM (red), and 30 μM TMG (dark blue). After 42 h growth, fluorescence microscopy was performed to determine the frequency of epigenetically ON cells. Shown are average fluorescence values per cell, with background fluorescence subtracted (Figure S1), for a representative set of 350 cells per treatment; each circle represents an individual cell.

To monitor the proportion of cells that are ON or OFF, we have replaced the *lacA* gene in the wild-type E. coli MG1655 chromosome (Table S1) with a *gfp* cassette, so that when the *lacZYA::gfp* transcript is expressed, β-galactosidase, galactoside permease, and green fluorescent protein are produced from the *lacZ*, *lacY*, and *gfp* genes, respectively (Figure 1A and Figure S1). The galactoside permease promotes the accumulation of the nonmetabolizable inducer thio-methylgalactoside (TMG). This permease induction and inducer accumulation provides the positive-feedback loop that throws the ON switch: permease transports the inducer of permease synthesis, TMG_in_ [14,27]. Our system exhibits bistability and hysteresis (history dependence), as was shown by Novick and Weiner [14] and Ozbudak et al. [27] for the *lac* system (Figure 1B and Figure S1). Moreover, Novick and Weiner [14] introduced the concept of a “maintenance” concentration of inducer, able to maintain the pre-existing state of cells, either maximally induced or uninduced. Cells already containing permease can accumulate enough inducer (TMG_in_) to maintain induction and so remain ON; uninduced cells, which lack permease, cannot accumulate TMG and so remain OFF (Figure 1A).

### Heritable Stochastic Switching in the *lac* Gene Network

*lac* repressor is rare (∼5–10 tetramers per cell) with the *lacI* gene transcribed about once per cell generation [28] and, due to intrinsic noise, repressor levels differ among isogenic cells and fluctuate with time within each cell [5]. A transient depletion of repressor within a cell will lead to a transient derepression of the operon and to a burst of *lacZYA::gfp* gene expression [10]. We reasoned that during growth of uninduced cells in a maintenance concentration of TMG, some cells would undergo stochastic events leading to permanent (DNA replication errors becoming mutations) or transient (transcription errors producing erroneous proteins) derepression of the *lac* operon. In the case of mutations, constitutive *lac* expression will ensue; in the case of transcription errors, the *lac* operon will be transiently derepressed but the subsequent appearance of permease will trigger the autocatalytic positive-feedback response so that the induced state will be heritably maintained, mimicking mutation [14,27] (Figure S6).

For our analysis, the MG1655 reporter strain was grown in a maintenance level of 6 μM TMG (Figure 1B; see also Figure 2B in Ozbudak et al. [27]). To determine the genetic (*lacI* or *lacO* ^c^) mutation frequency, we selected for colony-forming ability on agar plates containing phenyl-β-D-galactoside (Pgal) as the sole carbon source (Text S1). To determine the epigenetic-switch frequency, we measured the appearance of green cells by fluorescence microscopy (Figure S1). For wild-type MG1655, the frequency of epigenetically ON cells (0.3%) is ∼1,000-fold higher than the frequency of genetic *lac* constitutive mutations (Table 1 and Figure 1C). This result directly compares the frequency of permanent and transient errors in information transfer that lead to the same phenotype in the same system.

**Table 1:**
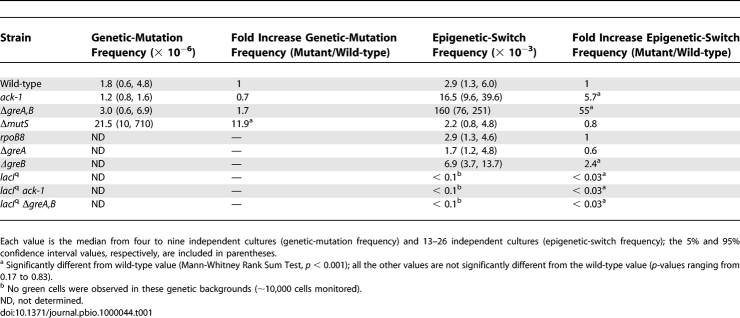
On the Origin of Heritable Phenotypic Change: Stochastic Switching Mediated by Permanent (DNA) and Transient (RNA) Errors in Information Transfer

### Maintenance in the *lac* System Depends upon the Low Levels of *lac* Repressor

Bistability in *lac* operon expression at 6 μM TMG (the maintenance effect [14]), depends upon the low native *lac* repressor concentration; when we introduced the *lacI* ^q^ up-promoter mutation into the reporter strain, causing a 10-fold increase in *lacI* transcription [28] and ∼50–100 repressor molecules per cell, bistability and the maintenance effect are abolished, regardless of genetic background (Table 1).

### Transcriptional Infidelity Increases Epigenetic Stochastic Switching

To determine whether the epigenetic-switch frequency is influenced by transcriptional fidelity, we deleted the *greA* and *greB* genes that encode auxiliary fidelity factors known from in vitro studies to facilitate the proofreading of misincorporations that arise in nascent mRNAs during transcription [29–31]. The Δ*greA* Δ*greB* double mutant increased the epigenetic-switch frequency 55-fold over the wild-type level, demonstrating a strong in vivo phenotype, but had no effect on the genetic-mutation frequency (Figure 1B and 1C, Table 1, and Figure S1). The Δ*greA* and Δ*greB* single mutants showed no large effect on the epigenetic-switch frequency, which is consistent with their common function in transcription fidelity (Table 1). To confirm that transcription infidelity is causing the increase in the epigenetic-switch frequency, we replaced the wild-type *rpoB* gene (encoding the β subunit of RNA polymerase, RNAP) with the *ack-1* allele that produces a RNAP known to increase ribonucleotide misincorporation both in vitro and in vivo [32]. The *ack-1* allele increased the epigenetic-switch frequency almost 6-fold compared with the wild-type level but had no effect on the genetic-mutation frequency (Table 1). Another *rpoB* allele*—rpoB8*, which shares many characteristics with *ack-1*, including rifampicin resistance [33], but is not known to affect transcription fidelity*—*had no significant effect on the epigenetic-switch frequency (Table 1). Finally, a Δ*mutS* deletion, which abolishes post-replicative mismatch repair and confers a DNA-mutator phenotype, had the complementary effect to the “RNA-mutators”: the genetic-mutation frequency was increased almost 12-fold over the wild-type level, but the epigenetic-switch frequency was unchanged. Therefore factors involved in the fidelity of RNA transcription affect the frequency of epigenetic heritable stochastic switching, while factors involved in the fidelity of DNA synthesis affect the frequency of heritable genetic stochastic events. While a decrease in transcriptional fidelity leads to an increase in intracellular noise that manifests itself in an increased switch frequency, it would be of equal interest to determine if an increase in transcriptional fidelity would lead to a decrease in intracellular noise and a decreased switch frequency. Until such an “anti-mutator” RNA polymerase is obtained, the role of transcription fidelity in the wild-type noise level remains suggestive; however, the analogous situation for DNA synthesis errors and their role in spontaneous mutation levels also exists.

### Transcriptional Infidelity and the Cellular Response to Inducer

Our results, presented in Table 1 and Figure 1C, show that a decrease in transcriptional fidelity increases heritable stochastic switching in a model bistable gene network. This is not because transcriptional infidelity makes the cell more responsive to 6-μM TMG, in that the Δ*greA* Δ*greB* strain, which exhibited the greatest increase in epigenetic-switch frequency, behaves the same as the wild-type strain with respect to sensing extracellular TMG: when ON cells from each strain are grown in the presence of decreasing concentrations of TMG, at 6-μM TMG, both wild-type and Δ*greA* Δ*greB* populations are fully ON (maintenance), and when the TMG concentration decreases beneath this point, the proportion of ON cells in the two strains falls in concert (Figure 1B). If Δ*greA* Δ*greB* cells were more responsive to TMG, then at lower concentrations than 6-μM TMG, a greater proportion of cells in this strain would remain fully ON compared with wild-type, which is not what we observe. We have also determined that these strains respond in an identical manner to isopropyl-thiogalactoside (IPTG), a similar gratuitous inducer to TMG but one that can more readily penetrate the cell (Figure S4). These results suggest that compromising the fidelity of transcription transiently reduces the concentration of functional proteins in a stochastic manner in rare individual cells, and that in the great majority of cells for the great majority of the time, there is no difference in the quantity or quality of the proteins being made in wild-type and Δ*greA* Δ*greB* cells. However, although Western blot analysis of *lac* repressor levels supports this contention that there is no general reduction in *lac* repressor levels in the Δ*greA* Δ*greB* background compared with wild-type (Figure S3) until repressor levels are quantified in individual cells in the wild-type and Δ*greA* Δ*greB* backgrounds, the possibility of slight differences in protein levels between the strains remains. Finally, β-galactosidase induction curves show that the Δ*greA* Δ*greB* strain and the *ack-1* strain are slightly dampened in their induction rates compared with wild-type, which would not be expected if transcriptional infidelity made the cell more responsive to TMG (Figure S2). Therefore, transcriptional infidelity does not make a cell more responsive to maintenance-level TMG.

It should also be noted that although the *ack-1* strain exhibits a slower doubling time than does the wild-type strain, a reduced growth rate, by itself, does not increase the stochastic switching frequency. Indeed, while the *rpoB8* control strain has a much longer doubling time than the RNA fidelity strains (Table S2) [33], it does not exhibit any increase in stochastic switching above the wild-type level (Table 1).

### Transcriptional Infidelity and the Secondary Channel of RNAP

The mechanism ensuring fidelity of transcription by RNAP and the biological consequences of transcription errors are not well understood. The consequences of increasing transcription errors have been obscured by the transient nature of mRNAs and a lack of appropriate experimental systems [34]. Our demonstration that a bistable switch can capture transient errors in transcription provides a ready approach to study and dissect in vivo the nature and the specificity of transcription fidelity.

Structures from prokaryotic and eukaryotic RNAPs have revealed a funnel-shaped pore, called the secondary channel, leading from the surface of the enzyme directly to the active site (Figure S5). This secondary channel is thought to be the major point of entry of ribonucleoside triphosphate substrates to the active site. We have now sequenced the DNA of the *ack-1* allele and reveal it to be a P564L substitution in the RNAP β subunit (Text S1) that is positioned at the strategic β turn, where the secondary channel opens directly onto the main channel and the active site. The only other RNAP fidelity mutation characterized in E. coli, an RpoB D675Y substitution [35], is also positioned at a β turn in the secondary channel but at the opposite end, on the surface or entry region of the secondary channel (Figure S5). Moreover, it is through this secondary channel that auxiliary fidelity factors (GreA, GreB, and TFIIS, the eukaryotic analogue of the Gre factors) access the active site to accomplish their proofreading activity. Misincorporations at the 3′ end of a nascent transcript have been proposed to arise by a misalignment mechanism common to all multisubunit RNAPs [36]. It has been proposed that a “product-assisted catalysis” occurs such that the 3′ misincorporated residue participates in its own excision via an intrinsic cleavage property of the RNAP that provides a proofreading fidelity mechanism [31]. This intrinsic proofreading is stimulated by auxiliary fidelity factors such as GreA and GreB [29–31] and TFIIS [31,37]. Therefore, residues composing the secondary channel, and auxiliary factors that reach the active site via the secondary channel, affect both transcriptional fidelity and the frequency of heritable stochastic switching. Additional mutational analysis of the secondary channel should provide insight into the mechanism of transcription fidelity.

### Transcription Errors Effecting Molecular Noise: A General Phenomenon?

In yeast, *trans*-acting mutations have been identified that contribute to noise and include the deletion of components of the SWI/SNF, INO80, and SAGA chromatin-remodeling complexes [38]. Recently, another study in yeast [39] has shown that mutations that impair uracil metabolism (*ura3*Δ), transcriptional elongation (*dst1*Δ or *spt4*Δ strains) or inactivate the Leo1p or Cdc73p subunits of the Paf1 complex can increase the level of noise in gene expression. Dst1 (TFIIS) and Spt4 are considered putative elongation factors, but it has recently been shown that neither factor affects the polymerase elongation rate [40]. The *dst1*Δ mutant does lack TFIIS activity, which is the analogous activity lost in Δ*greA* Δ*greB E. coli*; however, an altered transcriptional fidelity leading to increased noise was not considered for the *dst1*Δ mutant [39]. Since TFIIS has been demonstrated to be an auxiliary transcription fidelity factor [31,37,41,42], we propose that an increase in molecular noise resulting from a decrease in transcriptional fidelity may be a universal phenomenon, because it is observed in *dst1*Δ yeast and Δ*greA* Δ*greB E. coli*.

Since GreA is itself regulated, and GreA levels change in response to environmental cues [43], it is possible that the level of noise is under cellular control, and therefore subject to natural selection. Similar to a decrease in the fidelity of DNA polymerases under stressful conditions, which is a possible means to generate genetic diversity [44], a decrease in the fidelity of RNA transcription may also be viewed as a means to generate phenotypic diversity. Indeed, individuality in bacterial cells is now being considered as an evolutionary solution to the challenges of life in stochastic environments [45].

RNA errors occur frequently but are short-lived. Such transient errors can produce heritable epigenetic consequences if, for example, a positive-feedback loop is initiated. The idea that a transient transcription error can trigger a heritable epigenetic phenotypic change may also be considered in the origin of prion-like particles, where the altered protein acquires a dominant ability to alter its normal counterpart. Population heterogeneity and cell fate are being increasingly viewed as stochastic noise-driven processes [6,9,24,46]. Gene regulatory circuits are thought to control, exploit, or tolerate noise and so maintain order in the behavior and development of cells. Dysregulation in cell behavior is often due to mutation of control genes. Transient errors, by heritably altering epigenetic switch dynamics, may mimic mutation, and occur at much higher frequency than permanent errors. The resulting phenotypic heterogeneity may provide the raw material upon which selection can act with the subsequent evolution of novel cell characteristics during, for example, tumourigenesis: commitment to proliferation in mammalian cells is controlled by a positive-feedback loop, the “restriction point.” This critical bistable switch [19] may be triggered by transcription infidelity, and proliferation may become independent of growth stimulation as found in cancer cells. Thus “RNA mutators,” by introducing noise, should also be considered in the origin of altered or aberrant cell behavior.

## Materials and Methods

### Strain construction.

All strains are derived from the wild-type sequenced E. coli MG1655 strain (Table S1). The *gfp-cat* cassette was amplified from plasmid p1G (gift of Ariel Lindner, Université René Descartes, Paris, France) and integrated into the native *lac* operon by homologous recombination producing the *lacZYA::gfp* reporter. The *ack-l* allele (gift of Jonathan Gallant, University of Washington, Seattle, Washington, United States of America) and the Δ*greA*, Δ*greB* and Δ*mutS* deletion mutations (Keio Collection, Japan) were moved into MG1655 by P1 transduction.

### Growth media.

A small number of uninduced OFF cells (∼200) were grown for 42 h at 37 °C in minimal A salts plus MgSO_4_ with succinate as the sole carbon source, supplemented with varying amounts of TMG (to determine the genetic-mutation and epigenetic-switch frequencies, cells were grown at a maintenance level of 6-μM TMG) (Text S1). To determine the genetic-mutation frequency, we selected for colony forming ability on agar plates containing Pgal (75 μg/ml) as the sole carbon source. Only cells constitutively expressing β-galactosidase (*lacI^–^* and *lacO* ^c^ mutants) can form colonies on Pgal plates. To determine epigenetic-switch frequency, 1.0 ml of cells from the same subcultures used to determine genetic-mutation frequency was washed and concentrated 20-fold in minimal A salts buffer and 4 μl was used to prepare a microscope slide.

### Microscopy and image analysis.

Green fluorescence values of single cells were measured using a Zeiss HAL 100 inverted fluorescence microscope. Fields were acquired at 100× magnification with an EM-CCD camera (Hammamatsu). For each field, phase contrast and fluorescence (EGFP cube = Chroma, #41017) images were acquired. Image analysis was performed using AxioVision Rel. 4.6 (Zeiss). The program identified individual cells on phase contrast images and extracted pixel values from corresponding regions of fluorescence images to obtain the average fluorescence of each cell above the fluorescence background. The fluorescence levels of uninduced (OFF) cells are very similar to the fluorescence background.

## Supporting Information

Figure S1 Bistability in Single Cells The right panel shows overlayed green fluorescence and inverted phase-contrast images of Δ*greA* Δ*greB* cells that were initially uninduced (OFF) and then grown for 42 h in succinate minimal media with a maintenance concentration of TMG. In the left panel, each filled circle represents the average fluorescence (a.u.) of an individual cell; each open circle represents the background fluorescence of an equivalent area of slide that holds no cell. The autofluorescence of an uninduced (OFF) cell grown in succinate minimal media and maintenance level TMG does not rise much higher than background fluorescence. We determine an ON cell to be one that exhibits an average fluorescence that is greater than twice that of the background fluorescence.
(2.53 MB TIF)

Figure S2 Representative β-galactosidase Induction Curves Overnight minimal succinate cultures of wild-type (filled circles), *ack*-*1* (open circles), and Δ*greA* Δ*greB* (filled squares) cells were diluted to 0.1 OD600 in fresh minimal succinate media and grown to 0.2 OD600, and the cells were then induced by the addition of 1-mM TMG into the cultures; after various time points, β-galactosidase assays were performed [47]. Two independent experiments gave similar results.
(978 KB TIF)

Figure S3 *lac* Repressor Levels and GreA GreB Functional Status The levels of LacI were determined in MG1655 *lacI* ^q^ and MG1655 *lacI* ^q^ Δ*greA* Δ*greB* strains. *lacI* ^q^ derivative strains (which increase the level of *lacI* repressor about 10-fold) were used since the native level of *lac* repressor is beneath the level of Western blot sensitivity. Protein extracts were separated by SDS-PAGE (12%) and detected by Western blotting using an anti-LacI antibody; as a sensitivity control, protein extracts were also diluted 2-fold and exhibited a corresponding 2-fold difference in quantification. No difference in LacI repressor levels was observed between the two strains. Briefly, 25-ml cultures of MG1655 *lacI* ^q^ and MG1655 *lacI* ^q^ Δ*greA* Δ*greB* cells were grown at 37 °C to an OD600 of 0.4 in 0.2% succinate minimal A media from which 1-ml aliquots were spun down and resuspended in 100 μl of SDS loading buffer. Samples (15 μl) of undiluted or 1:2 dilutions were then run on 12% polyacrylamide gels and analyzed by Western blotting. Western blots were performed with primary antibodies against LacI (1:5000 dilution of anti-LacI rabbit polyclonal IgG [48], generously provided by Prof. Hiroji Aiba, Nagoya University, Japan) and then revealed with secondary goat anti-rabbit IgG (1:2000 dilution of Alex Fluor 647, Invitrogen). The PVDF membrane was scanned with a Typhoon Trio according to the manufacturer (GE).
(1.06 MB TIF)

Figure S4 IPTG Concentration and Maintenance of the Fully Induced Cell Cells that were originally ON were subcultured and grown in media containing various concentrations of IPTG. Each value is the average ± SD from 3–9 independent cultures (wild-type, filled circle; Δ*greA* Δ*greB*, open circle; *lacI* ^q^, filled square; *lacI* ^q^ Δ*greA* Δ*greB*, open square).
(1.19 MB TIF)

Figure S5 The Tertiary Context of Characterized E. coli RNA Polymerase Fidelity Mutants A schematic representation of T. aquaticus RNAP is presented [49]. P564 (*ack*-*1*) and D675 [35] residues correspond to T. aquaticus residues P444 and D554 (shown in red). Top panel: the arrows highlight transcription fidelity mutations that alter amino acids composing the secondary channel (dark green ribbon: β subunit; light green ribbon: β' subunit; blue ribbons: α subunits). Bottom panel: close-up view of the RNAP secondary channel. The substituted amino acids residues are depicted as red space-filling balls.
(2.27 MB TIF)

Figure S6 The Capture and Heritable Maintenance of Transient *lac* Operon Induction in E. coli Cells TMG is a gratuitous, nonmetabolizable inducer of the *lac* operon; maintenance-level TMG maintains *lac* operon induction in induced cells, but does not induce the *lac* operon in uninduced cells.
**Capture**. Induced cells arise by expression of the galactoside transport system, initiating an autocatalytic cycle of induction since the permease can transport an inducer for its own synthesis; therefore, when cells are grown in the presence of maintenance level TMG, transient events that precipitate induction of the *lac* operon are captured and heritably maintained and exhibit a clonal nature.
**Selection**. β-galactosidase is required for the utilization of Pgal as a carbon source, but Pgal does not induce synthesis of the enzyme. However, Pgal can be converted into an inducer by β-galactosidase (albeit inefficiently relative to lactose); therefore, growth on Pgal is autocatalytic depending upon the initial level of β-galactosidase [50]: only cells already expressing the *lac* operon will grow on these selection plates (*lacI* or *lacO* ^c^ constitutive cells or induced wild-type operon ON cells, “I^−^ transient” cells) Note that for induced cells to form a colony on Pgal plates, the Pgal concentration has to be at least 8-fold higher than the concentration found in mutation selection plates (600 μg/ml versus 75 μg/ml).
**Deadaptation**. When induced wild-type cells (*lac* operon ON) are grown in the absence of inducer, they undergo deadaptation (eventually *lac* operon OFF). Therefore, by picking colonies from the initial Pgal plates onto minimal media glucose plates, all cells will grow, but while *lacI*/*lacO* ^c^ mutant cells will continue to constitutively express the *lac* operon, “I^−^ transient” cells will undergo deadaptation and eventually become uninduced (repressed) under this non-maintenance condition.
**Discrimination**. After deadaptation on glucose plates, the colony grids are replica plated to Pgal selective plates and to 5-bromo-4-chloro-3-indolyl-β-D-galactoside (Xgal) indicator plates. This challenge will distinguish *lacI*/*lacO* ^c^ mutants (growth on Pgal; blue patches on Xgal) from “I^−^ transient cells” (no growth on Pgal; white patches on Xgal). Xgal is a non-inducing chromogenic substrate of β-galactosidase. Therefore, by combining maintenance during the growth of the culture and selection for *lac* expression on agar plates (Pgal), both *lacI*/*lacO* ^c^ mutations and captured “I^−^ transient” events are clonal, selectable, distinguishable, and subject to fluctuation analysis. Briefly, an overnight MG1655 culture, inoculated from a single colony and grown in minimal A medium and 0.2% succinate, was diluted and ∼200 cells were seeded to new tubes containing fresh medium, with 6-μM TMG, and shaken at 37 °C for 42 h. Dilutions of the subcultures were spread on selection plates (minimal A medium supplemented with 0.06% Pgal and 1.5% purified agar) and minimal A glucose plates and incubated for 48 h at 37 °C. Pgal is a substrate of β-galactosidase and can act as a carbon source; other trace carbon sources were removed from the Pgal plates by spreading 5 × 10^7^ CH256 scavenger bacteria 20 h before use. The average epigenetic frequency observed for wild-type MG1655 cells was 2.6 × 10^−3^ (20 independent cultures). This is similar to the average frequency obtained using single-cell analysis, 3.0 × 10^−3^ (26 independent cultures). After deadaptation, the great majority of colonies tested (97%; *n* = 469) comprised wild-type cells, and therefore formed white patches on Xgal plates and did not form patches on Pgal plates. This is because the autocatalytic cycle is broken, and once broken, this autocatalytic cycle does not become fully restored when replated onto Pgal or Xgal plates. It should be noted that in the original Novick and Weiner study [14], when a culture was started from a single uninduced cell and grown for over 100 generations in succinate medium with a maintenance level of TMG, that culture never attains more than 0.5% the level of β-galactosidase synthesis compared to a culture which was initiated from a single induced cell. Of course, since enzyme induction in this case is an all-or-none phenomenon, this means that the proportion of fully ON cells is <0.5% in the otherwise OFF population. All of these results demonstrate the heritability of the ON state in maintenance TMG conditions, and the frequency of the stochastic switch from OFF to ON is also consistent between these studies and methodologies.
(996 KB TIF)

Table S1 Bacterial Strains (76 KB DOC)

Table S2 E. coli Doubling Times in Minimal A Salts Plus Succinate Medium (26 KB DOC)

Text S1 Supplementary Methods (79 KB DOC)

## Abbreviations

Pgal: phenyl-β-D-galactoside
RNAP: RNA polymerase
TMG: thio-methylgalactoside
