# Supplementary figures and images for: Transcriptional Infidelity Promotes Heritable Phenotypic Change in a Bistable Gene Network
Source: PLoS Biol. 2009 Feb 24;7(2):e1000044. doi: 10.1371/journal.pbio.1000044 (PMC2652393; doi:10.1371/journal.pbio.1000044)

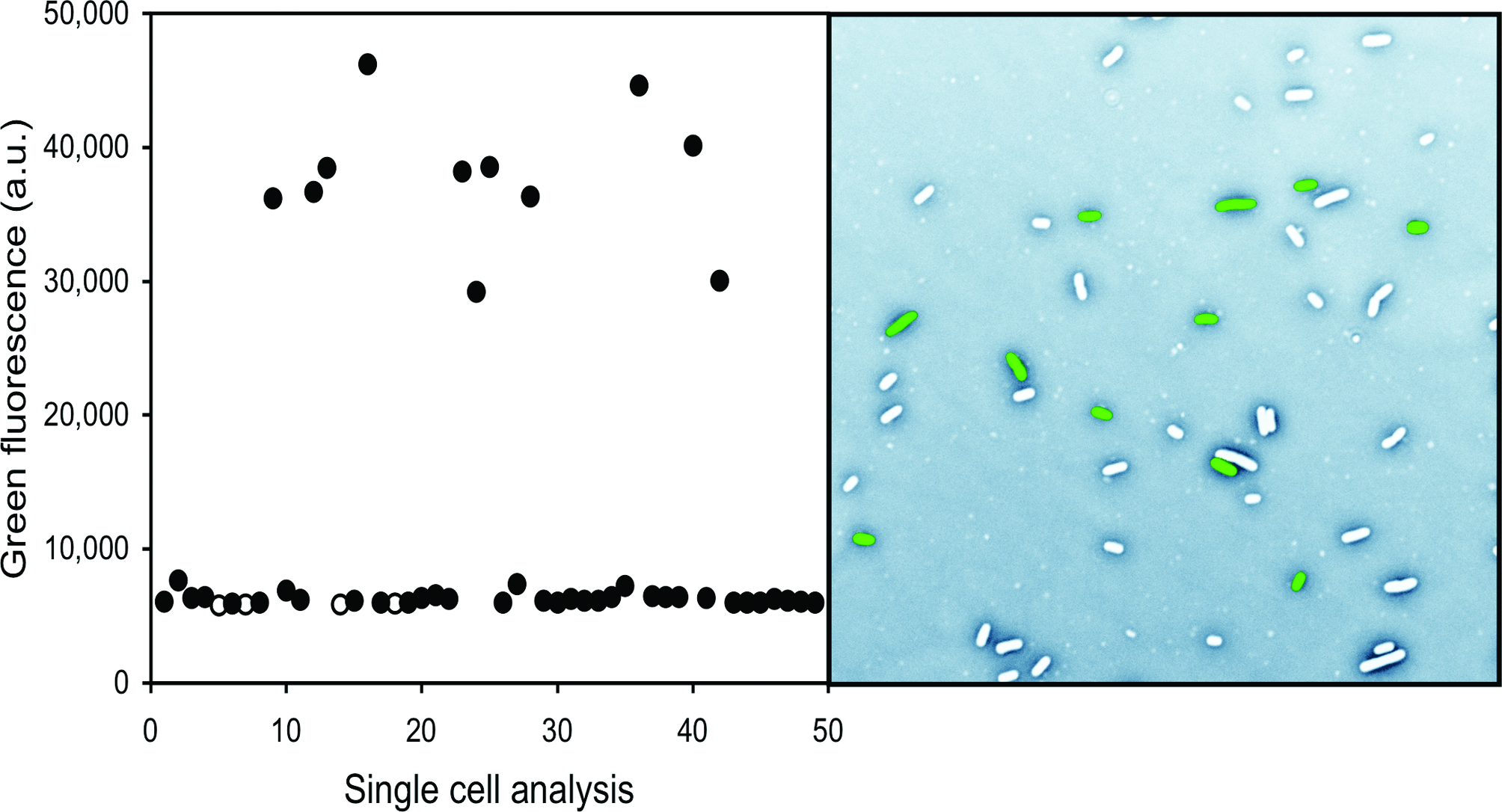

Supplement: Figure S1 — The right panel shows overlayed green fluorescence and inverted phase-contrast images of ΔgreA ΔgreB cells that were initially uninduced (OFF) and then grown for 42 h in succinate minimal media with a maintenance concentration of TMG. In the left panel, each filled circle represents the average fluorescence (a.u.) of an individual cell; each open circle represents the background fluorescence of an equivalent area of slide that holds no cell. The autofluorescence of an uninduced (OFF) cell grown in succinate minimal media and maintenance level TMG does not rise much higher than background fluorescence. We determine an ON cell to be one that exhibits an average fluorescence that is greater than twice that of the background fluorescence. (2.53 MB TIF) [file pbio.1000044.sg001.tif]

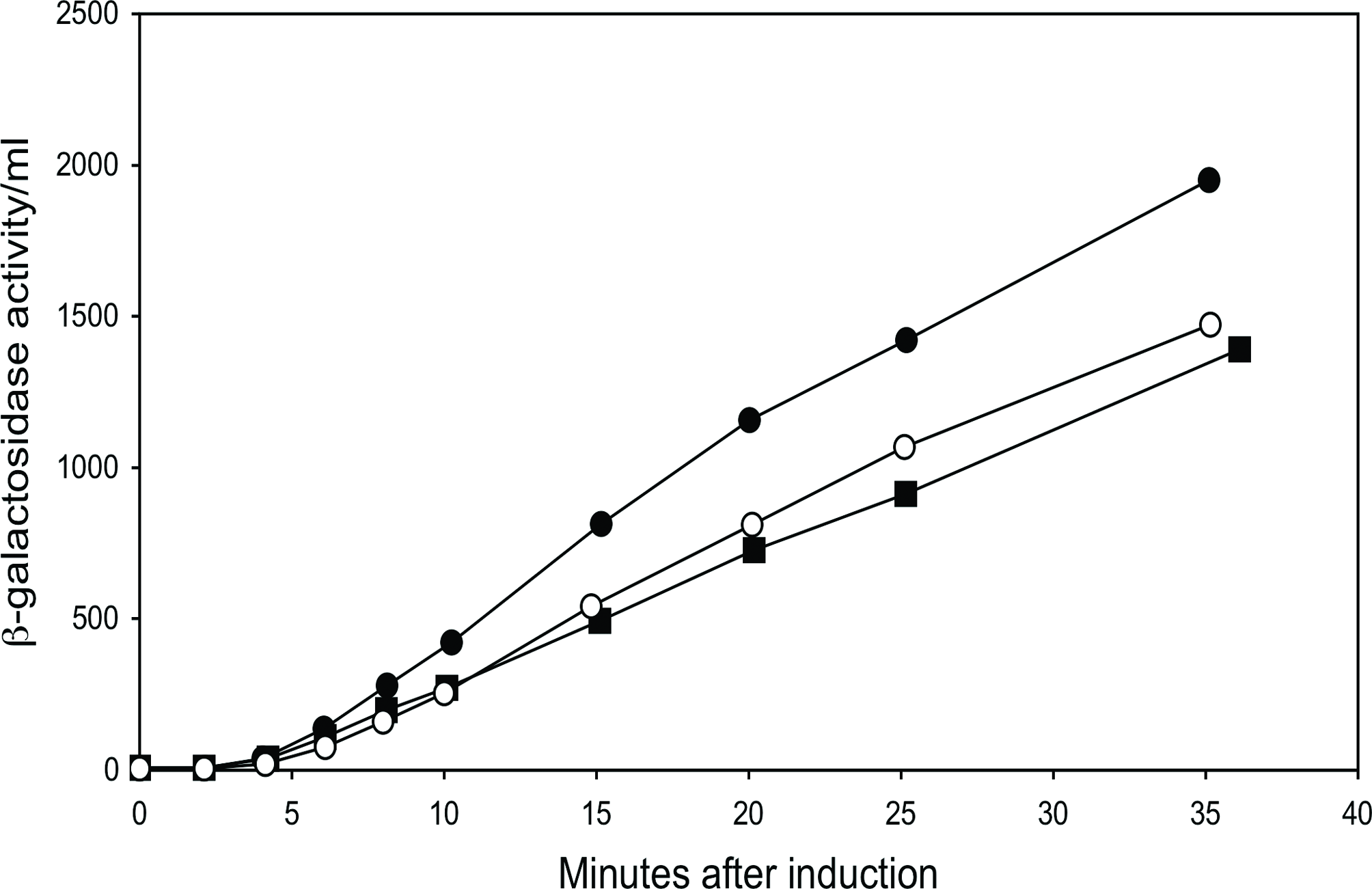

Supplement: Figure S2 — Overnight minimal succinate cultures of wild-type (filled circles), ack-1 (open circles), and ΔgreA ΔgreB (filled squares) cells were diluted to 0.1 OD600 in fresh minimal succinate media and grown to 0.2 OD600, and the cells were then induced by the addition of 1-mM TMG into the cultures; after various time points, β-galactosidase assays were performed [47]. Two independent experiments gave similar results. (978 KB TIF) [file pbio.1000044.sg002.tif]

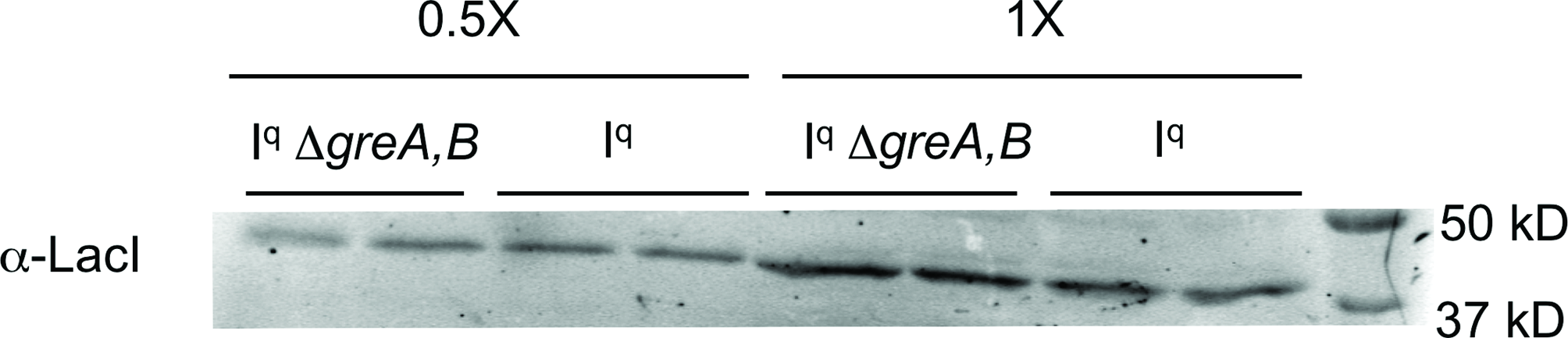

Supplement: Figure S3 — The levels of LacI were determined in MG1655 lacI q and MG1655 lacI q ΔgreA ΔgreB strains. lacI q derivative strains (which increase the level of lacI repressor about 10-fold) were used since the native level of lac repressor is beneath the level of Western blot sensitivity. Protein extracts were separated by SDS-PAGE (12%) and detected by Western blotting using an anti-LacI antibody; as a sensitivity control, protein extracts were also diluted 2-fold and exhibited a corresponding 2-fold difference in quantification. No difference in LacI repressor levels was observed between the two strains. Briefly, 25-ml cultures of MG1655 lacI q and MG1655 lacI q ΔgreA ΔgreB cells were grown at 37 °C to an OD600 of 0.4 in 0.2% succinate minimal A media from which 1-ml aliquots were spun down and resuspended in 100 μl of SDS loading buffer. Samples (15 μl) of undiluted or 1:2 dilutions were then run on 12% polyacrylamide gels and analyzed by Western blotting. Western blots were performed with primary antibodies against LacI (1:5000 dilution of anti-LacI rabbit polyclonal IgG [48], generously provided by Prof. Hiroji Aiba, Nagoya University, Japan) and then revealed with secondary goat anti-rabbit IgG (1:2000 dilution of Alex Fluor 647, Invitrogen). The PVDF membrane was scanned with a Typhoon Trio according to the manufacturer (GE). (1.06 MB TIF) [file pbio.1000044.sg003.tif]

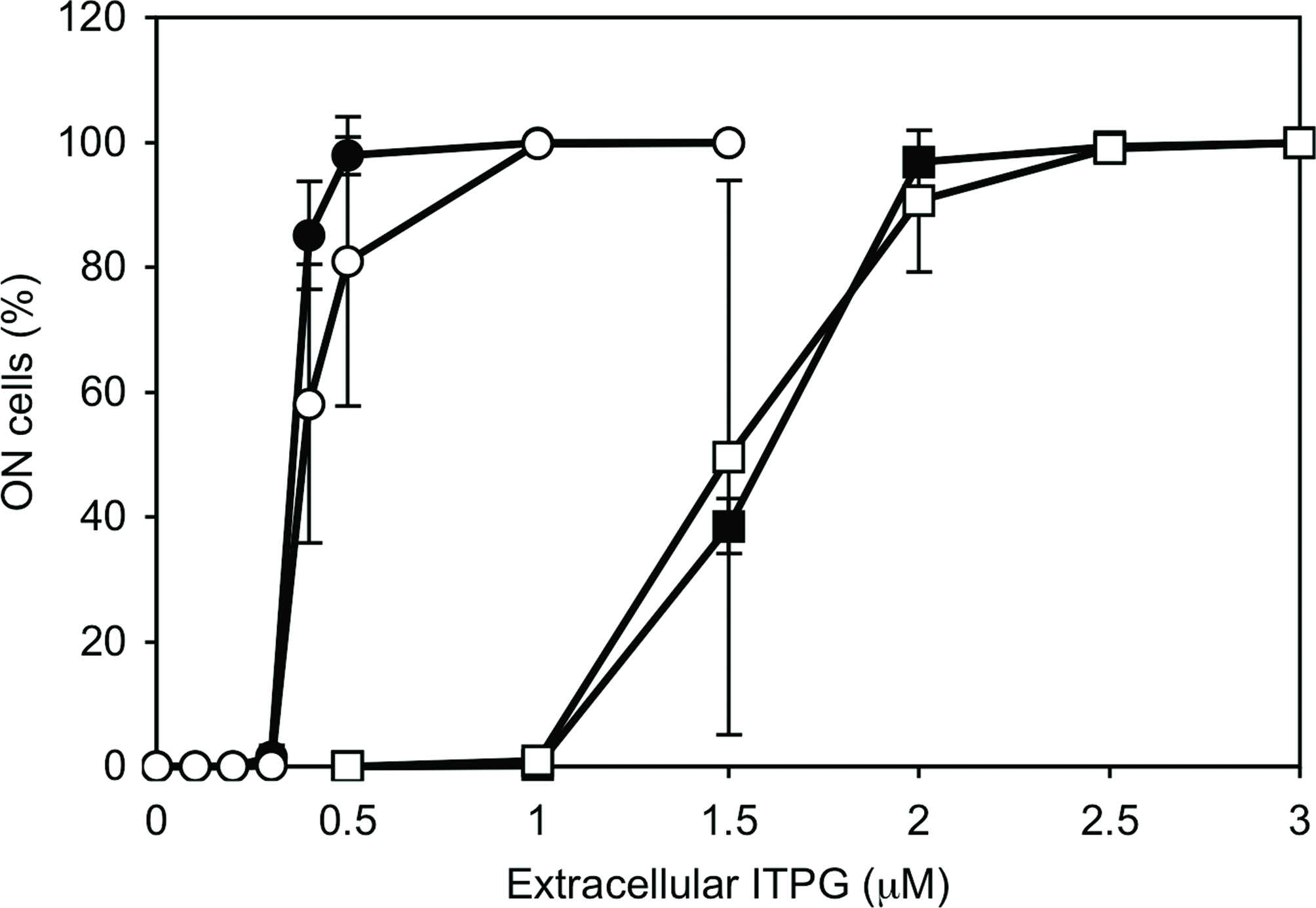

Supplement: Figure S4 — Cells that were originally ON were subcultured and grown in media containing various concentrations of IPTG. Each value is the average ± SD from 3–9 independent cultures (wild-type, filled circle; ΔgreA ΔgreB, open circle; lacI q, filled square; lacI q ΔgreA ΔgreB, open square). (1.19 MB TIF) [file pbio.1000044.sg004.tif]

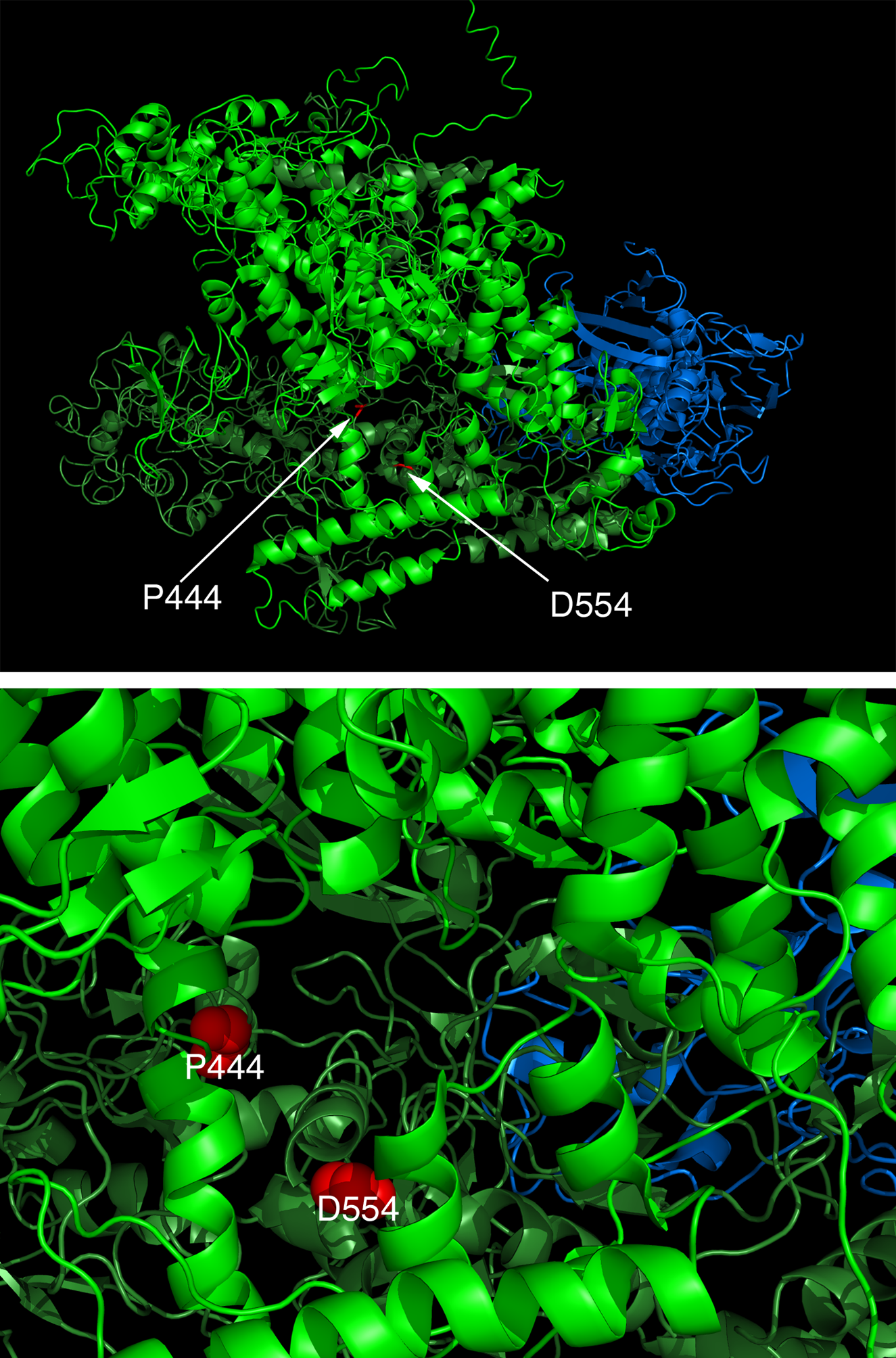

Supplement: Figure S5 — A schematic representation of T. aquaticus RNAP is presented [49]. P564 (ack-1) and D675 [35] residues correspond to T. aquaticus residues P444 and D554 (shown in red). Top panel: the arrows highlight transcription fidelity mutations that alter amino acids composing the secondary channel (dark green ribbon: β subunit; light green ribbon: β' subunit; blue ribbons: α subunits). Bottom panel: close-up view of the RNAP secondary channel. The substituted amino acids residues are depicted as red space-filling balls. (2.27 MB TIF) [file pbio.1000044.sg005.tif]

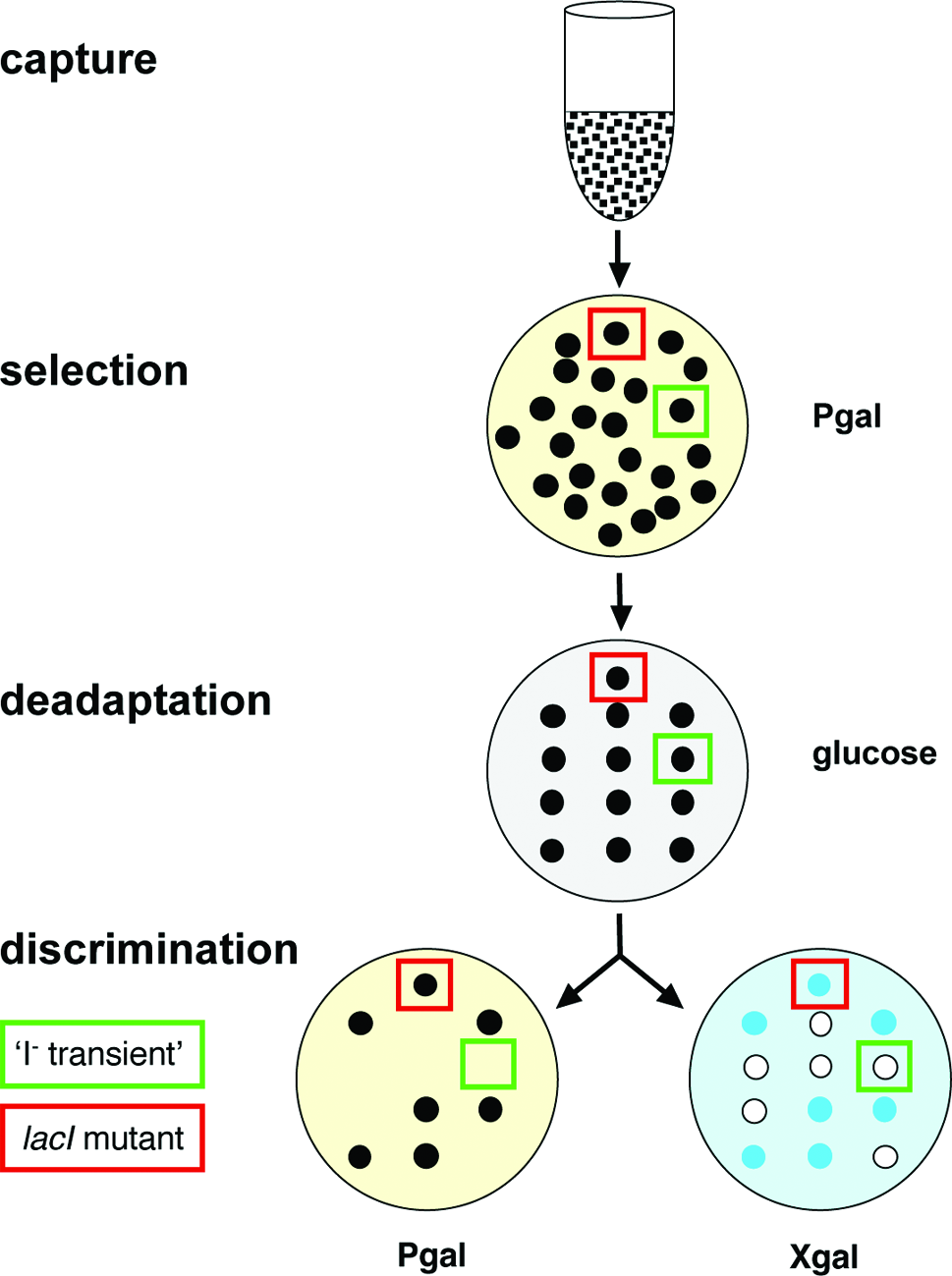

Supplement: Figure S6 — TMG is a gratuitous, nonmetabolizable inducer of the lac operon; maintenance-level TMG maintains lac operon induction in induced cells, but does not induce the lac operon in uninduced cells. Capture. Induced cells arise by expression of the galactoside transport system, initiating an autocatalytic cycle of induction since the permease can transport an inducer for its own synthesis; therefore, when cells are grown in the presence of maintenance level TMG, transient events that precipitate induction of the lac operon are captured and heritably maintained and exhibit a clonal nature. Selection. β-galactosidase is required for the utilization of Pgal as a carbon source, but Pgal does not induce synthesis of the enzyme. However, Pgal can be converted into an inducer by β-galactosidase (albeit inefficiently relative to lactose); therefore, growth on Pgal is autocatalytic depending upon the initial level of β-galactosidase [50]: only cells already expressing the lac operon will grow on these selection plates (lacI or lacO c constitutive cells or induced wild-type operon ON cells, “I− transient” cells) Note that for induced cells to form a colony on Pgal plates, the Pgal concentration has to be at least 8-fold higher than the concentration found in mutation selection plates (600 μg/ml versus 75 μg/ml). Deadaptation. When induced wild-type cells (lac operon ON) are grown in the absence of inducer, they undergo deadaptation (eventually lac operon OFF). Therefore, by picking colonies from the initial Pgal plates onto minimal media glucose plates, all cells will grow, but while lacI/lacO c mutant cells will continue to constitutively express the lac operon, “I− transient” cells will undergo deadaptation and eventually become uninduced (repressed) under this non-maintenance condition. Discrimination. After deadaptation on glucose plates, the colony grids are replica plated to Pgal selective plates and to 5-bromo-4-chloro-3-indolyl-β-D-galactoside (Xgal) indicator plates. This ch [file pbio.1000044.sg006.tif]
