# Supplementary material for: Transcriptional Infidelity Promotes Heritable Phenotypic Change in a Bistable Gene Network
Source: PLoS Biol. 2009 Feb 24;7(2):e1000044. doi: 10.1371/journal.pbio.1000044 (PMC2652393; doi:10.1371/journal.pbio.1000044)
Supplement: Table S1 — (76 KB DOC) [file pbio.1000044.st001.doc]

**Table S1**

Bacterial strains

| Name | Genotype | Reference or Source |
| --- | --- | --- |
| MG1655 | F^-^ l^-^ *ilvG rfb-50 rph-1*, sequenced wild-type K12 | Lab. stock |
| CH256 | MG1655 *lacIpoZΔ*(*Mlu*) | Lab. stock |
| CH393 | MG1655 Δ*greA_FRT_* | MG1655 x P1(JW3148) |
| CH395 | MG1655 Δ*greB_FRT_* | MG1655 x P1(JW3369) |
| CH495 | MG1655 Δ*greA_FRT_* Δ*greB_FRT_* | CH393 x P1(JW3369) |
| CH458 | MG1655 *lacZYA::gfp-cat* | Recombineering [1]; this study |
| CH503 | MG1655 Δ*mutS_kan_ lacZYA::gfp-cat* | CH458 x P1(JW2703) |
| CH568 | MG1655 Δ*greA_FRT_* Δ*greB_FRT_ lacZYA::gfp-cat* | CH495 x P1(CH458) |
| CH1348 | MG1655 Δ*greA_FRT_* *lacZYA::gfp-cat* | CH393 x P1(CH458) |
| CH1350 | MG1655 Δ*greB_FRT_ lacZYA::gfp-cat* | CH395 x P1(CH458) |
| CP79-U118 rif^r,b^ | *thr leu his arg thi relA-2 lacZ-U118 ack-1* | Jonathan Gallant |
| CAG5052 | λ^-^ *relA1 spoT1 metB1 btuB3191::Tn10* | CGSC |
| CP79-U118 rif^r,b^ tet^r^ | *thr leu his arg thi relA-2 lacZ-U118 ack-1 btuB3191::Tn10* | CP79-U118 rif^r,b^ x P1(CAG5052) |
| CH515 | MG1655 *ack-1 btuB3191::Tn10 lacZYA::gfp-cat* | CH458 x P1(CP79-U118 rif^r,b^ tet^r^) |
| CF2013 | MG1655 *rpoB8 btuB::Tn10* | Michael Cashel |
| CH1417 | MG1655 *rpoB8 btuB::Tn10 lacZYA::gfp-cat* | CF2013 x P1(CH458) |
| NCM514 | MG1655 *I^q^*Δ*zah_cat_* | CGSC |
| CH1118 | MG1655 *lacZYA::gfp-FRT* | CH458 *cat* flipped out |
| CH1143 | MG1655 *I^q^*Δ*zah_cat_ lacZYA::gfp-FRT* | CH1118 x P1(NCM514) |
| CH1528 | MG1655 *I^q^*Δ*zah_cat_ lacZYA::gfp-FRT ack-1 btuB3191::Tn10* | CH1143 x P1(CP79-U118 rif^r,b^ tet^r^) |
| CH1388 | MG1655 *I^q^_cat_* Δ*greA_FRT_* Δ*greB_FRT_* | CH495 x P1(NCM514) |
| CH1415 | MG1655 *I^q^_FRT_* Δ*greA_FRT_* Δ*greB_FRT_* | CH1388 *cat* flipped out |
| CH1466 | MG1655 *I^q^_FRT_* Δ*greA_FRT_* Δ*greB_FRT_ lacZYA::gfp-cat* | CH1415 x P1(CH458) |

1. Datsenko KA, Wanner BL (2000) One-step inactivation of chromosomal genes in Escherichia coli K-12 using PCR products. Proc Natl Acad Sci U S A 97: 6640-6645.
