# Supplementary material for: Transcriptional Infidelity Promotes Heritable Phenotypic Change in a Bistable Gene Network
Source: PLoS Biol. 2009 Feb 24;7(2):e1000044. doi: 10.1371/journal.pbio.1000044 (PMC2652393; doi:10.1371/journal.pbio.1000044)
Supplement: Table S2 — (26 KB DOC) [file pbio.1000044.st002.doc]

**Table S2**

*E. coli* Doubling Times in Minimal A Salts plus Succinate Medium.

| Strain | Doubling Time (min) |
| --- | --- |
| wild-type | 91 |
| Δ*greA* Δ*greB* | 89 |
| *ack-1* | 108 |
| *rpoB8* | 158 |
