# Supplementary material for: Transcriptional Infidelity Promotes Heritable Phenotypic Change in a Bistable Gene Network
Source: PLoS Biol. 2009 Feb 24;7(2):e1000044. doi: 10.1371/journal.pbio.1000044 (PMC2652393; doi:10.1371/journal.pbio.1000044)
Supplement: Text S1 — (79 KB DOC) [file pbio.1000044.sd001.doc]

**Supplementary Methods**

**Bacterial strains**

All the strains that were analyzed in this study are derived from the wild-type sequenced *E. coli* MG1655 strain (Table S1). Manipulation of the MG1655 genome was accomplished by standard methodologies [1,2]. *E. coli* strains JW3148, JW3369, JW2703 are from the Keio collection of precisely defined gene knockouts [3] in which the gene in question (*greA, greB, mutS*, respectively) is replaced by a kanamycin resistance gene that is bounded by FRT sequences and thereby the *kan* gene can be removed by a plasmid-borne FLP recombinase resulting in an FRT scar [1]. The *lacI^q^* up-promoter mutation is tightly linked to a *cat* gene bounded by FRT sequences. The *cat* gene in the *gfp-cat* cassette is also bounded by FRT sequences and is tightly linked to the *gfp* gene that was introduced into the *lac* operon replacing the *lacA* gene and thereby putting *gfp* expression under the regulation of the *lac* repressor (see below). A *btuB::Tn10* allele (gift of Michael Cashel, NIH) was introduced into strain CP79-U118 rif^r,b^ (gift of Jonathan Gallant, U. of Washington) by P1 transduction; the *btuB::Tn10* and *ack-1* alleles are 70% co-transducible. The *ack-1* allele was introduced into MG1655 by selecting for tetracycline resistance and then scoring for rifampicin resistance, a phenotype conferred by the *ack-1* allele of the *rpoB* gene. Therefore, all mutant alleles/gene cassettes were moved into new bacterial strains by P1 transduction [2] and selected by resistance to appropriate antibiotics (chloramphenicol, 12.5 μg/ml; tetracycline, 10 μg/ml; kanamycin, 30 μg/ml); the subsequent elimination of drug resistance markers to allow further strain construction was accomplished by FLP recombinase [1].

**Construction of *the lacZYA::gfp-cat* strain**

The *gfp-cat* cassette was amplified from plasmid p1G (gift of Ariel Lindner, Hôpital Necker, Paris France) by PCR using primers OC191 LacA-gfp (5’-gcgccttatccgaccaacatatcataacggagtgatcgcattaagaaggagatatacatatggc-3’) and OC192 LacA-gfp (5’-ttaaactgacgattcaactttataatctttgaaataatagtggtcgagattttcaggagc-3’). Using the method of Wanner [1] this 2.4 kb PCR fragment was incorporated into the *lac* operon such that the *gfp* gene now replaces the *lacA* gene at the start codon of *lacA* and *gfp* becomes the ultimate sequence in the *lacZYA::gfp* transcript (Figure 1A). The *cat* gene is under separate regulation and does not form part of the *lacZYA::gfp* transcript. Importantly, all the known sequences that are involved in normal *lac* operon regulation (including operators *O_1_*, *O_2_* and *O_3_*) are intact and functional in the *lacZYA::gfp-cat* strain.

**DNA sequencing the *ack-1* allele**

The *ack-1* allele has previously been genetically mapped to a small region encompassing amino acids 565 and 576 of the *rpoB* gene [4]. We PCR amplified a 258 bp fragment of strain CP79-U118 rif^r,b^ including this region using primers rpoBinternalverL (5’-aaacgtcgtatctccgcact-3’) and rpoBinternalverR (5’cctgggcgataacgtagttg-3’). DNA sequence analysis of the amplified fragment revealed that the *ack-1* mutation corresponds to a CCT to CTT transition resulting in a Pro to Leu amino acid substitution at position 564. When the amino acid sequence of *E.coli rpoB* is superimposed upon the solved three-dimensional structure of the RpoB protein of *T. aquaticus* [5], the *E. coli* P564L residue corresponds to *T. aquaticus* P444 and is positioned at a β-turn where the secondary channel opens onto the main channel and the active site (see Figure S3). It is of interest that the only other RNA polymerase fidelity mutation that has been characterized in *E. coli*, a GAC to TAC base substitution that results in a Asp to Tyr amino acid substitution at residue 675 [6] is also positioned at a β-turn forming the secondary channel, but at the opposite end, at the surface or entry region of the secondary channel; *E. coli* D675 corresponds to *T. aquaticus* D554.

**Growth conditions and media**

Cells were grown at 37^o^C in minimal A salts [2] plus MgSO_4_ (1 mM) with succinate (0.2%) as the sole carbon source, supplemented with varying amounts of TMG. To demonstrate hysteresis and bistability in *lac* operon expression in single cells, an overnight bacterial culture of the strain of interest carrying the *lacZYA::gfp* construct, inoculated from a single colony and grown in minimal succinate media, was diluted 1:3 in fresh medium with (ON culture) or without 1 mM TMG (OFF culture) and shaken at 37^0^C for 7 h. After this induction period, the two cultures were individually diluted and ~200 cells were seeded to new tubes containing fresh medium that contained varying amounts of TMG, and shaken at 37^0^C for 42 h. To determine the percentage of cells that were induced for *lac* operon expression (ON cells), 1.0 ml of cells from the subcultures was washed and concentrated 20-fold in minimal A salts buffer and 4 μl was used to prepare a microscope slide (Figure 1B; Figure S1).

To determine epigenetic and genetic switch frequencies, an overnight bacterial culture of the strain of interest carrying the *lacZYA::gfp* construct, inoculated from a single colony and grown in minimal succinate media, was diluted and ~200 cells were seeded to new tubes containing fresh medium, with a maintenance level of 6 μM TMG, and shaken at 37^0^C for 42 h. To determine genetic-mutation frequencies, dilutions of the subcultures were spread on selection plates (minimal A medium supplemented with 75 μg/ml Pgal and 1.5% purified agar) and minimal A glucose (0.2%) plates and incubated for 2-3 days at 37^0^C. Pgal is a substrate of β-galactosidase and can act as a carbon source but does not induce *lac* operon expression, therefore only cells constitutively expressing β-galactosidase (*lacI^-^* and *lacO^c^* mutants) can form colonies on Pgal plates [2]; other trace carbon sources were removed from the Pgal plates by spreading 5x10^7^ CH256 scavenger bacteria 20 h before use. To determine epigenetic-switch frequency, 1.0 ml of cells from the same subcultures used to determine genetic frequency was washed and concentrated 20-fold in minimal A salts buffer and 4 μl was used to prepare a microscope slide (Figure 1B; Figure S1).

1. Datsenko KA, Wanner BL (2000) One-step inactivation of chromosomal genes in *Escherichia coli* K-12 using PCR products. Proc Natl Acad Sci U S A 97: 6640-6645.

2. Miller JH (1992) A short course in bacterial genetics : a laboratory manual and handbook for *Escherichia coli* and related bacteria. Plainview, N.Y.: Cold Spring Harbor Laboratory Press.

3. Baba T, Ara T, Hasegawa M, Takai Y, Okumura Y, et al. (2006) Construction of *Escherichia coli* K-12 in-frame, single-gene knockout mutants: the Keio collection. Mol Syst Biol 2: 2006 0008.

4. Libby RT, Nelson JL, Calvo JM, Gallant JA (1989) Transcriptional proofreading in *Escherichia coli*. Embo J 8: 3153-3158.

5. Zhang G, Campbell EA, Minakhin L, Richter C, Severinov K, et al. (1999) Crystal structure of *Thermus aquaticus* core RNA polymerase at 3.3 A resolution. Cell 98: 811-824.

6. Holmes SF, Santangelo TJ, Cunningham CK, Roberts JW, Erie DA (2006) Kinetic Investigation of *Escherichia coli* RNA Polymerase Mutants That Influence Nucleotide Discrimination and Transcription Fidelity. J Biol Chem 281: 18677-18683.
